# A Reconstructed Common Ancestor of the Fatty Acid Photo‐decarboxylase Clade Shows Photo‐decarboxylation Activity and Increased Thermostability

**DOI:** 10.1002/cbic.202000851

**Published:** 2021-03-31

**Authors:** Yue Sun, Elia Calderini, Robert Kourist

**Affiliations:** ^1^ Institute of Molecular Biotechnology Graz University of Technology Petersgasse 14 8010 Graz Austria

**Keywords:** ancestral sequence reconstruction, biofuels, fatty acids, photo-biocatalysis, photo-decarboxylation, thermostability

## Abstract

Light‐dependent enzymes are a rare type of biocatalyst with high potential for research and biotechnology. A recently discovered fatty acid photo‐decarboxylase from *Chlorella variabilis* NC64A (CvFAP) converts fatty acids to the corresponding hydrocarbons only when irradiated with blue light (400 to 520 nm). To expand the available catalytic diversity for fatty acid decarboxylation, we reconstructed possible ancestral decarboxylases from a set of 12 extant sequences that were classified under the fatty acid decarboxylases clade within the glucose‐methanol choline (GMC) oxidoreductase family. One of the resurrected enzymes (ANC1) showed activity in the decarboxylation of fatty acids, showing that the clade indeed contains several photo‐decarboxylases. ANC1 has a 15 °C higher melting temperature (*T*_m_) than the extant CvFAP. Its production yielded 12‐fold more protein than this wild type decarboxylase, which offers practical advantages for the biochemical investigation of this photoenzyme. Homology modelling revealed amino acid substitutions to more hydrophilic residues at the surface and shorter flexible loops compared to the wild type. Using ancestral sequence reconstruction, we have expanded the existing pool of confirmed fatty acid photo‐decarboxylases, providing access to a more robust catalyst for further development via directed evolution.

## Graphical abstract

**Enzyme resurrection strikes again**: Ancestral sequence reconstruction was applied to a putative photo‐decarboxylases clade. One common ancestor showed up to 12‐fold production yield, a 15 °C increase in thermostability and up to 20‐fold improvement in residual activity when incubated at 50 °C. Ancestral sequence reconstruction proved to be a valuable tool to introduce valid mutations to a little understood fatty acid photo‐decarboxylase.

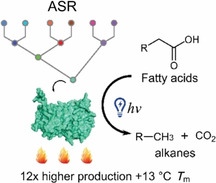

## Introduction

Light is essential for sustaining life on earth: It is essential to generate carbohydrates and oxygen in plants and microalgae.^[1]^ Photochemical reactions are fundamental for photosynthesis, vision and the biosynthesis of vitamin D,^[2]^ among the most relevant. Nevertheless, if we do not consider photosynthesis, a surprisingly small number of photoenzymatic reactions have been identified, these being the photolyases involved in the repair of DNA damage^[3]^ and the photo‐biocatalytic reaction for the reduction of C=C double bonds by protochlorophyllide reductases.^[4]^ The successful exploitation of coupling photosystem with biocatalysis spun into the new research field photo‐biocatalysis.[^5^, ^6^] Recently, a new type of light‐dependent enzymes has been discovered in the microalgae *C. variabilis*: the fatty acid photo‐decarboxylase (CvFAP).^[7]^ The enzyme belongs to the glucose‐methanol choline (GMC) oxidoreductase family, which comprises a wide range of oxidases and dehydrogenases.

While previously known oxidative fatty acid decarboxylases such as OleT^[8]^ from *Jeotgallicoccus* and UndA/B^[9]^ from *Pseudomonas* produce terminal olefins; CvFAP can produce industrially attractive compounds with low consumption of energy using renewable starting material. Furthermore, it can not only convert carboxylic acids into hydrocarbons but also can recognize cis/trans isomers and stereoisomers.^[10]^ Additionally, the range of compounds that can be converted to alkanes could be increased in cascade reactions. For example, Hollmann's group reported the combination of CvFAP with lipases to convert waste material to more valuable products.[^11^, ^12^] The photoenzymatic cascades comprise CvFAP to transform unsaturated fatty acid in alkenes and subsequently an hydratase converts them into alcohol. Here, CvFAP lacks the chloroplast targeting sequence at the enzyme's N terminus (sCvFAP), as it was shown to increase both conversion and enzyme's production.^[13]^

The conversion of (waste) carboxylic acids and oils into biofuels and corresponding synthetic applications has been the focus of research for decades.[^14^, ^15^]

Unfortunately, synthetic applications of CvFAP are limited by its relatively poor production in *Escherichia coli*, its light stability and its sensitivity to solvents.[^11^, ^16^, ^17^] All this results in a limited product formation, which cannot pay off the effort for the production of the enzyme. On top of the low stability, production yields remain low, which is an obstacle for both the enzyme's biochemical characterization and industrial application – even though the purification under red light was beneficial.[^10^, ^11^, ^13^] Engineering of enzymes is a proficient method to increase their operational stability.[^18^, ^19^, ^20^] Despite its recent discovery, several groups reported enzyme engineering studies to improve CvFAP's substrate spectrum and enantioselectivity.[^10^, ^21^] While changes in catalytic activity or selectivity can be achieved with a small number of mutations, improvements in stability and production in bacteria often require a higher number of mutations. So far, predicting combinations of many mutations is out of our understanding or current computational power. Ancestral sequence reconstruction (ASR) is the probabilistic reconstruction of ancient protein sequences based on extant genes. It has emerged as a proficient method to produce functional proteins that keep the aspects of the protein family.[^22^, ^23^] Reconstructed enzymes may not only have a broader substrate range compared to their modern descendants,^[24]^ but often show improved thermostability and solvent tolerance.[^25^, ^26^] Therefore, we hypothesized that the common ancestor in the FAP clade identified by Sorigue et al.^[7]^ would still show photo‐decarboxylation but with improved stability and/or soluble production in *E. coli* as demonstrated previously.[^27^, ^28^] To verify this, we set out to reconstruct three possible ancestors of this clade and investigate their photo‐decarboxylative activity.

## Results and Discussion

### ASR of photo‐decarboxylases produces functional enzymes

**Figure 1:**
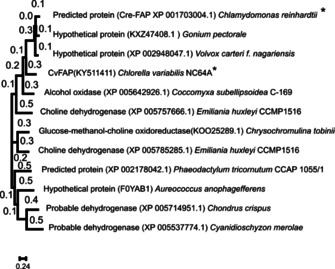
Phylogenetic relationship of the 12 sequences of extant FAP enzymes used to reconstruct the ancestral sequences; * enzymes with confirmed photo‐decarboxylation activity at the time of the reconstruction.

The typical first step of any ancestral sequence reconstruction is the collection of a set of recent homologous sequences. The most straightforward way to gather a large number of sequences is a similarity search. The major downfall is that public databases contain more sequences than needed for a successful ASR. In our case, a second risk was present as CvFAP is a member of the glucose‐methanol‐choline (GMC) superfamily. Thus, a BLAST search would produce a highly unbalanced dataset with the risk to lose the light‐dependent activity and produce an ancestral oxidoreductase. Giving the extremely rare frequency of photoenzymes, we decided to only include 12 sequences that were classified in the fatty acid photo‐decarboxylases clade by Sorigue and co‐workers where only two enzymes had confirmed photo‐decarboxylation activity at the time of the reconstruction, lately during the experimental characterization more photo‐decarboxylases were experimentally verified (Figure 1).[^7^, ^29^] All sequences belong to the algal genus. As ancestral enzymes have shown before to have an improved soluble production in *E. coli*, the expectation was that the same could be achieved for the photo‐decarboxylases.[^27^, ^28^]

**Figure 2:**
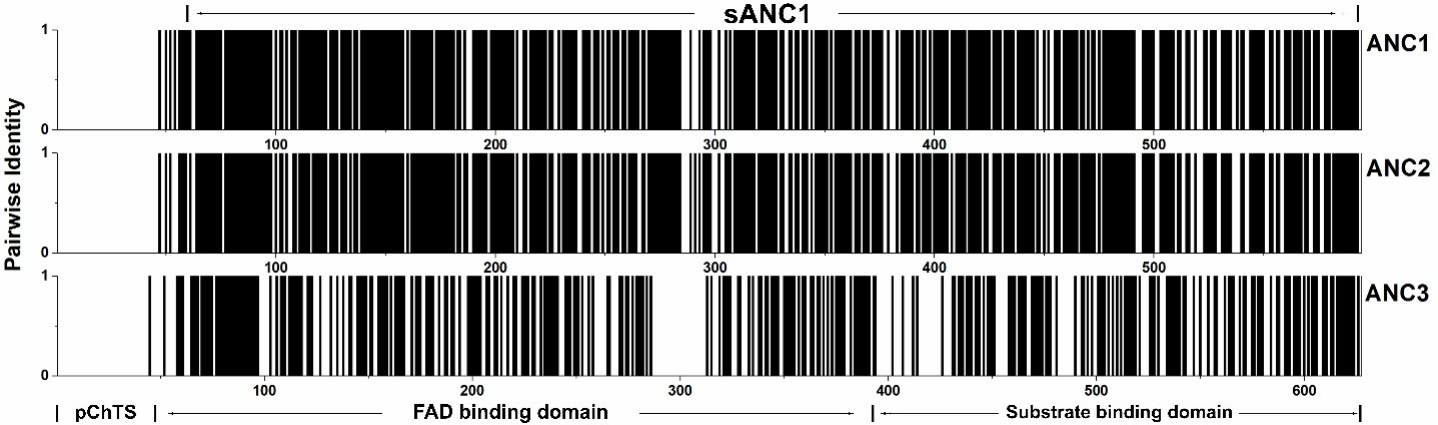
Pairwise identity between each ancestor and sCvFAP. The residue number is given on the *x*‐axis; the *y*‐axis denotes pairwise identity at each site. Black bars denote the pairwise identity of 100 % at each site. The portion of ANC1 corresponding to sANC1 is highlighted at the top of the figure. (pChTS: predicted chloroplast targeting sequence.)

Aware of the limitation of our dataset, we performed three different reconstructions named ANC1, ANC2 and ANC3 using different sequence alignment tools and evolutionary models to increase the probability to obtain an active variant. The sequences of the node 0 elements from the three different reconstructions were ordered as codon‐optimized synthetic genes. The sequence identity with the wild type was determined to be 76 % for ANC1 and ANC2 and 54 % for ANC3, respectively (a multiple sequence alignment is available in Figure S8 in the Supporting Information). Figure 2 visually illustrates how ANC1 and ANC2 are more similar to WT (more black bars) whereas ANC3 has a lower sequence identity (more white bars). Despite the differences, they all share the conserved amino acid positions that characterize FAPs: C432, R451 and A576 (numbering corresponding to CvFAP).[^29^, ^30^] A576 is possibly one of the most striking difference between FAPs and other members of the GMC superfamily. At this position, most members of the GMC superfamily have a histidine.^[30]^

### ANC1 showed better production yield and stability

So far sCvFAP production in *E. coli* has shown limited yields of soluble protein in cell‐free extract and, particularly, low enzyme production after purification.^[17]^ In general, efforts to regulate the solubility of the protein have included the use of weak promoters, low cultivation temperature during protein production, modified growth media, and fusion with solubility enhancing tags.[^31^, ^32^, ^33^] A recently published study demonstrated that higher expression levels could be achieved by removing the predicted chloroplast targeting sequence in CvFAP^[11]^ and that loss of catalytic activity could be reduced by performing the *in vitro* purification under red light.^[17]^

**Figure 3:**
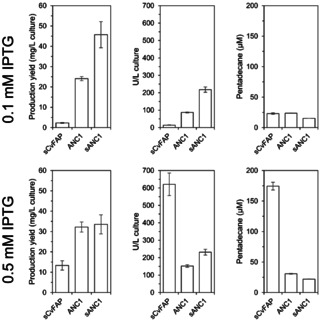
Comparison of sCvFAP, ANC1 and sANC1 production under two different IPTG concentrations. The plots on the left report production yield as mg/L culture, the plots in the centre report units per litre culture, and the plots on the right show pentadecane production from palmitic acid as substrate after 1 h using 4 μM of purified enzymes. Units are given per mg of FAD‐loaded enzyme.

**Figure 4:**
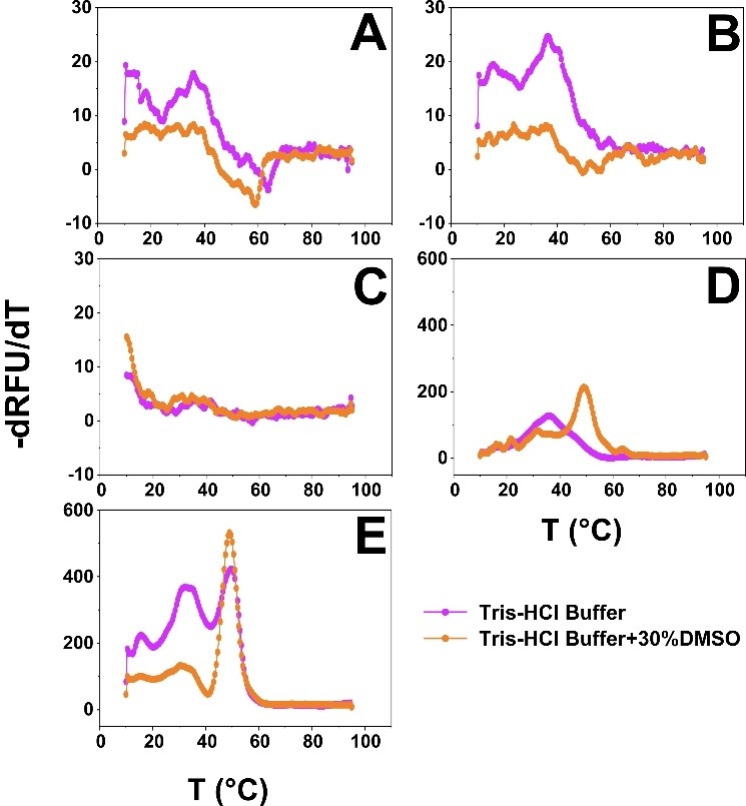
First derivative curves of the melting temperature curves obtained in fluorescence measurements. A) sCvFAP produced in *E. coli* TOP10 (pBAD‐sCvFAP); B) sCvFAP produced in *E. coli* BL21(DE3) (pASK‐sCvFAP); C) sCvFAP produced in *E. coli* BL21(DE3) (pET28a‐sCvFAP); D) ANC1 produced in *E. coli* BL21(DE3) (pET28a‐ANC1); E) sANC1 produced in *E. coli* BL21(DE3) (pET28a‐ sANC1).

As already shown by several studies, the concentration of the inductor IPTG is crucial for soluble production of the CvFAP variant lacking the chloroplast targeting sequence (sCvFAP) when using the T7 system.[^34^, ^35^, ^36^] This was confirmed with an increase of the production yield from 2 mg/L culture (with 0.1 mM IPTG) to 13 mg/L culture (with 0.5 mM IPTG). Interestingly, a similar purification yield (Table S2) was achieved using low copy plasmid such as pASK or pBAD, whose gene expression is under control of anhydrotetracycline or arabinose, respectively (Table S2, Figure S2). Testing the expression of sCvFAP under different promoters was crucial to obtain a reliable melting temperature curve (Figure 4). Unfortunately, ANC2 and ANC3 showed extremely low soluble production (Figure S1) and no activity was detected (data not shown). Therefore, the higher expression level and loss of catalytic activity for sCvFAP is not only influenced by in vitro purification but also by the intracellular protein folding (e. g., removing the predicted chloroplast targeting sequence, promoter strength or decreasing IPTG concentration; Table S2, Figure S2). In contrast, using the same approaches for ANC1 (e. g., removing the predicted chloroplast targeting sequence or increasing IPTG concentration) further increased soluble expression and production yield of ANC1. After one‐step purification, sANC1 showed a 20‐fold higher production yield compared to sCvFAP when using 0.1 mM IPTG, and still a three‐fold higher yield when using 0.5 mM (Figure 3). Here, the final yield of purified ANC1 was 32.2±2.5 mg/L culture, sANC1 reached 33.3±4.7 mg/L culture, whereas sCvFAP purification only reached 13.3±2.3 mg/L culture (Figure 3, bottom left). For the three variants we determined the FAD content to be approximatively 22 % for sCvFAP, 42 % for ANC1 and 30 % for sANC1. An higher FAD loading is possibly another benefit of the enzyme purification under red light as this resulted in 69 % FAD loaded enzyme.^[17]^ Nevertheless, FAD integration seems to be a slow process as supplementing FAD at different ratios (1 : 10, 1 : 1 and 10 : 1) did not show significant improvements (Figure S7).

Surprisingly, a higher concentration of IPTG resulted in an extremely high improvement in sCvFAP total and specific activity (Figure 3, Table S3); however, the thermostability decreased significantly (see below). This resulted in a 40‐fold improvement in the volumetric activity of cell‐free extracts compared to expression with 0.1 mM IPTG. For ANC1, the volumetric activity only increased 1.7 times whereas sANC1 showed comparable performance across both conditions. It appears that ANC1 and sANC1 production is more consistent at different IPTG concentrations. In contrast, the functional production of sCvFAP is favoured at higher IPTG concentrations.

The poor solubility of free fatty acids in water limit the load of the substrate, and furthermore, a co‐solvent is always needed to increase the substrate solubility further. Organic solvents can interact with the hydrophobic patches of proteins perturbing their folding/unfolding equilibrium in aqueous solutions.^[37]^ Deep eutectic solvents (DESs) have often been used as an alternative to organic solvents.[^38^, ^39^] DES and DES/water mixtures have been shown to partially preserve enzyme activity leading to improved performances compared to similar setups using organic solvents.^[40]^ Several attempts were also made in biocatalysis where DES have been used as (co‐)solvents resulting in increased substrate solubility and enzyme stability.[^41^, ^42^]

In a previous work, switching to a DES system allowed us to increase the substrate concentration from 10 mM to 300 mM using a phenolic acid decarboxylase as biocatalyst.^[42]^ Thus, we envisioned that DES could be beneficial.

Despite both sCvFAP and ANC1 could perform photo‐decarboxylation in DES, no considerable improvement was observed compared to the aqueous system with 30 % DMSO as co‐solvent. It is interesting to note that the activity of sCvFAP in ChCl:Gly DES decreased. ANC1 activity was nine times higher in the DES/water mixture than in aqueous solution but did not exceed that of sCvFAP under the same conditions (Table 1).

**Table 1:** Conversion of palmitic acid to pentadecane^[a]^ using different mixtures of DES and water.

	Reaction medium	sCvFAP^[b]^	ANC1^[a]^
1	DMSO (30 %)	24 %	1.6 %
2	DES/H_2_O^[c]^ (50 %)	16 %	16 %
3	DES/H_2_O^[c]^ (60 %)	9 %	6 %
4	DES/H_2_O^[c]^ (70 %)	8 %	4 %

[a] Reaction conditions: Palmitic acid=10 mM, purified enzymes=10 μM, Reaction time=20 h. [b] Conversion determined by GC‐FID. [c] DES ChCl:Gly in a 1 : 2 molar ratio mixed in different ratios in H_2_O.

### Influence of the putative chloroplast targeting sequence on the properties of ANC1

As reported earlier, removing the predicted chloroplast targeting sequence in sCvFAP increased the solubility and production yield significantly, and could slightly improve catalytic activity.^[11]^ Even though the ASR already resulted in a shorter N terminus compared to wild type, we attempted to further improve ANC1 production and activity by investigating whether the resurrected enzyme presented the predicted chloroplast targeting sequence and if removing it would have similar effects observed for wild type.

Thus, the shorter variant comprising residues 62–623 and thus lacking the predicted chloroplast targeting sequence of ANC1 (sANC1) was recombinantly produced in *E. coli* BL21(DE3) and purified. As seen for ANC1, sANC1 showed similar soluble overproduction, production yield (Figure S1) and slightly better decarboxylation activity with palmitic acid as the substrate compared to the extended version ANC1 (Figure 3).

In previous studies, sCvFAP showed different substrate preference for oleic acid, linoleic acid, and saturated counterpart stearic acid as a substrate.^[11]^ Therefore, we selected these three substrates of ANC1 and sANC1 to investigate if the resurrected enzymes displayed a different preference compared to wild type. Interestingly, ANC1 showed no activity for the unsaturated substrates whereas, sANC1 showed conversion for all three but with a high preference for the unsaturated counterpart (Table 2). It appears that the chloroplast targeting sequence influences ANC1 substrate preference as increasing the amount of enzyme did not show an increased conversion for the unsaturated substrate in ANC1 (data not shown). Furthermore, ANC1 and sANC1 with their increased thermostability are possibly more rigid than wild type; thus we believe that unsaturated fatty acids are less likely to fit in the active tunnel since they are also more rigid due to the double bond compared to their saturated counterparts.^[43]^ This might contribute to the higher ANC1 chemoselectivity towards unsaturated fatty acids compared to wild type.

**Table 2:** Decarboxylation of ANC1 and sANC1 of various fatty acid substrates compared to wild‐type sCvFAP (5 mM substrate, 3 h reaction time).

Fatty acid	Product [μM]		
	CvFAP	ANC1	sANC1
C18:0	1146.9	128.1	278.8
C18 : 1 Δ9	651.2	0	25.9
C18 : 2 Δ9,12	29.6	0	16.5

### ANC1 and sANC1 have higher T_m_ and thermostability

In many cases, ancestral proteins are more thermostable than modern proteins. This is often explained by a higher average earth temperature in prehistoric times.^[19]^ We anticipated that our ancestral proteins would show higher thermostability compared to sCvFAP. Therefore, we determined the melting temperatures (*T*_m_) for sCvFAP and the ancestral proteins using the ThermoFAD method.^[44]^ This method relies on the fact that the flavin‐containing proteins display different fluorescent properties between the folded and denatured state. In particular, during the denaturation of flavoproteins, the secondary and tertiary structure of the protein is disrupted, and interactions with the flavin break down. The released free flavin usually results in a large increase in fluorescence at 530 nm.^[45]^ The denaturation of sCvFAP^[17]^ and its resurrected ancestor ANC1 yielded a very pronounced decreasing fluorescence intensity. This was in contrast to the known increasing fluorescence observed with a flavin‐dependent monooxygenase CahJ that was used as control (Figure S3).^[44]^ Thus, in this case, the *T*_m_ is identified by plotting the negative of the first derivative of the fluorescence emission as a function of temperature (‐d*F*/d*T*).^[44]^ At first, the fluorescent signal of sCvFAP was low and presented undefined peaks. To resolve this, we performed the thermoFAD experiment with sCvFAP expressed under different promoters to obtain more defined melting temperature curves of sCvFAP for better comparison with the profiles observed for ANC1 and sANC1 (Figure 4). In particular, we used pASK and pBAD where the heterologous gene is under the control of anhydrotetracycline (Ptet) and arabinose (araBp).

In all cases, a profile with two peaks in the derivative curve was found, indicating that two distinct domains unfold at different temperatures. This is in agreement with observations from Sorigue et al.,^[7]^ who described a structure with two distinct domains, one for binding FAD and one for the substrate, respectively. We hypothesized that for the expression of the sCvFAP gene under the control of the T7 promoter, we obtained a mixed population of correctly folded and unfolded sCvFAP that resulted in the undefined profile (Figure 4C). Producing sCvFAP from vectors with a weaker promoter gave similar production yields, but resulted in a more homogeneous enzyme population with defined melting temperature profiles (Figure 4A and B).

Here, ANC1 and sANC1 showed a significantly higher melting temperature for both domains with an increase of 15 and 13 °C, respectively (Table 3). Furthermore, the addition of 30 % DMSO (Figure 4, orange curves) seems to accelerate the denaturation of the second peak, as a faster denaturation results in a higher ‐d*F*/d*T*. Possibly, the second peak is the substrate‐binding domain as it should be more hydrophobic to interact with fatty acids. Thus, while interacting more easily with DMSO, it unfolds at a faster rate.

**Table 3:** Comparison of *T*_m_ of both peaks determined by ThermoFAD for sCvFAP, ANC1 and sANC1 in aqueous buffer with or without 30 % DMSO.

Expression	*T*_m_[°C]	
vector	Tris ⋅ HCl buffer	Tris ⋅ HCl buffer+30 %DMSO
pBAD‐sCvFAP	14/36	18/36
pASK‐sCvFAP	16/36.5	24/35.5
pET28a‐sCvFAP	n.d.	n.d.
pET28a‐ANC1	36/44	31/49
pET28a‐sANC1	36/49.5	31/49

n.d. not determined.

**Figure 5:**
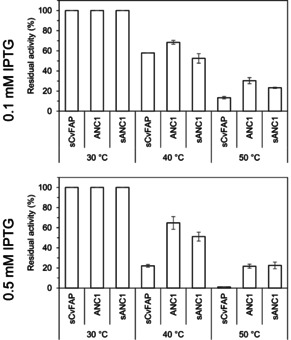
Comparison of sCvFAP, ANC1 and sANC1 residual activities at different temperatures and IPTG concentrations. The photoenzymatic decarboxylation reactions were catalysed by sCvFAP, ANC1, and sANC1 produced using different IPTG concentrations (0.1 and 0.5 mM). Purified enzymes (4 μM) were incubated at different temperatures for 10 min, and then the reactions were performed with gentle magnetic stirring at 30 °C in a total volume of 200 μL Tris**⋅**HCl buffer (pH 8.5, 100 mM) containing 30 % DMSO.

To validate this data, we further determined the thermostability of ANC1, sANC1, and sCvFAP by measuring the residual activity at 30 °C after incubating the purified enzymes at a range of temperatures (Figure 5). The plots confirm that ANC1 and sANC1 are more thermostable photo‐decarboxylases compared to sCvFAP. Again, different IPTG concentrations in protein production had a more significant effect on sCvFAP compared to the resurrected enzymes. In particular, sCvFAP produced with 0.5 mM IPTG seems to be approximately three times less thermostable compared to sCvFAP produced with 0.1 mM IPTG. For some reason, a higher concentration of IPTG contributes to producing a more active enzyme, which is far more prone to aggregation and thermal denaturation (Figure 5). ANC1 and sANC1 seem more robust to changes in expression conditions and maintained similar residual activity at both IPTG concentrations. It is striking that when produced with 0.1 mM concentration ANC1 showed a twofold improvement compared to wild‐type in residual activity at 50 °C, whereas it displayed almost 20‐fold improvement when produced at 0.5 mM at 50 °C compared to wild type produced under the same conditions. All in all, the higher thermostability determined with the ThermoFAD method could be validated with activity measurements confirming that ANC1 and sANC1 are significantly more thermostable than the wild‐type enzyme.

Unfortunately, the increase in thermostability did not translate in higher resistance to light inactivation. Here we found that ANC1 is slightly more photosensitive than wild type (Figure S6).

Lakavath and co‐workers described the radical based photoinactivation as an intrinsic property of sCvFAP which is due to localised modification of protein residues around FAD and in the active site.^[17]^ It is unclear why the chloroplast targeting sequence should have such effect on photoinactivation giving that ANC1 and sANC1 share the same active site and FAD biding domain, however elucidating this was out of the scope of this study.

### Homology modelling and structural considerations

**Figure 6:**
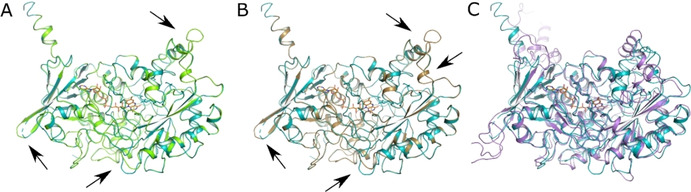
Homology models of ancestors A) ANC1 (green), B) ANC2 (sand) and C) ANC3 (purple) superimposed on the sCvFAP structure (turquoise; PDB ID: 5NCC). Black arrows highlight the main differences visible between WT and the ancestors’ homology models. Images generated in PyMOL 2.3.5.

The availability of the three‐dimensional structure of the enzyme from *C. variabilis* (PDB ID: 5NCC) made it possible to generate homology models for all ancestors using the Robetta server and try to infer possible reasons for the higher thermostability and production.^[46]^ Aligning the homology models to the experimental crystal structures showed few structural variations, primarily characterized by shorter loop regions, whereas ANC3 seems to have more differences compared to sCvFAP structure (Figure 6).

As the homology models were built based on the wild type structure, no dramatic structural changes were visible, the most striking difference, especially in the active ANC1, is the length of a few flexible loops which seem to be shorter. Shorter flexible loops in the ancestor might have contributed to a higher enzyme's rigidity resulting in increased thermostability.

**Figure 7:**
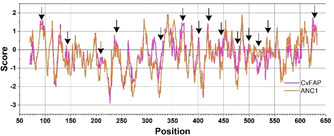
ProtScale hydrophobicity graph for sCvFAP (pink) and ANC1 (orange). A positive score is associated with higher hydrophobicity. Arrows designate the surface residues where appreciable changes are visible.

One proven method to increase enzyme stability is to increase the packing at the enzyme's core with hydrophobic interaction and to increase the hydrophilicity of surface residues for better interaction with aqueous systems.[^19^, ^47^] Thus, we investigated whether we could observe significant changes in surface residues. As anticipated, most of the substitutions on the outer surface of ANC1 were towards more hydrophilic amino acids (Figure 7). In contrast, we could not identify variants in the protein core that may explain the improvement in the production yield of ANC1 without compromising the functional activity.

## Conclusion

The photo‐decarboxylase from *C. variabilis* CvFAP shows unique activity in the conversion of fatty acids to alkanes. The successful reconstruction of the common ancestor ANC1 from a clade containing CvFAP, CreFAP and 10 enzymes that had been so far annotated as oxidases confirms the assumption that all members of this clade might be photo‐decarboxylases. This assumption was later further confirmed by Moulin and co‐workers that confirmed FAP activity for additional enzymes.^[29]^ Two additional ancestors could not be characterized due to problems with the soluble production after expression of their genes in *E. coli*. ANC1 shows higher production of soluble protein in *E. coli*, which could be further increased by N‐terminal truncation. Moreover, we were pleased to observe that purification yields were higher than with the extant decarboxylase, which is an important advantage for the biochemical characterization of the enzyme. Interestingly, the N‐terminal truncation influenced the substrate spectrum as well, which is difficult to explain with the currently available homology models of ANC1. It is a common notion that ancestral enzymes often show improved thermostability. Indeed, besides improved production yield, ANC1 and its shorter version sANC1 showed a remarkable increase in melting temperature of 15 and 13 °C for the two protein domains, respectively.

Homology modelling and analysis of surface residues highlighted that most of the surface residues substitutions were towards more hydrophilic residues and most flexible loops in ANC1 were a few residues shorter. Possibly, this contributed to the higher thermostability observed for ANC1 by decreasing its flexibility. Common protein engineering methods do not take into account the length of flexible loops, and often thermostability is achieved by mutagenesis; however, it seems that optimizing loop length could be a viable option when more stability is desired without significant changes in catalytic activity or substrate scope.

All in all, ancestral sequence reconstruction proved to be a valuable tool even with a limited amount of extant sequences giving the extremely rare nature of photoenzymes. Despite the trade‐off in activity, ANC1 and sANC1 have superior handling compared to wild type; thus it could also be used as a scaffold for further improvements and for industrial applications.

## Experimental Section

Ancestral sequence reconstruction (ASR) of the photo‐decarboxylase was done using the online tool GRASP.^[48]^ In particular, the 12 sequences classified in the photo‐decarboxylase clade were submitted to the stated different sequence alignment tools (see below) available from the EMBL‐EBI website. Subsequently, the alignments were inspected for possible artefacts or sequences out place and afterwards the phylogenetic trees were inferred by maximum likelihood using RAxML. Finally, different evolutionary models readily available in the GRASP suite were selected to increase the probability to obtain an active variant. All algorithms were performed under default settings. More in detail ANC1 was generated using T‐coffee^[49]^ for the sequence alignment, and the JTT^[50]^ evolutionary model. ANC2 was generated by changing the evolutionary model to LG,^[51]^ whereas ANC3 was generated by using MAFFT^[52]^ for the sequence alignment and the JTT evolutionary model.

The ancestral FAPs were modelled using Robetta.^[46]^ The surface residues selection and Images of protein structures were prepared using PyMOL.^[53]^ The hydropathic scale were computed and represented using ProtScale.[^47^, ^54^]

Sequences, plasmid, strains, and growth conditions: All DNA sequences used are collected in Table S1. All the ancestral sequence reconstructions were synthesized and cloned into pET28a with *C*‐terminal His tag. *E. coli* DH5α was used for the propagation of plasmids. *E. coli* BL21(DE3) was used for the high‐level expression of the recombinant photo‐decarboxylases.

Protein expression and purification: For detailed analysis, the ancestral variants, *E. coli* BL21(DE3) harbouring the appropriate plasmid were grown at 37 °C in LB supplemented with 50 μg/mL of kanamycin, until an OD_600 nm_ of approximately 0.8 was reached. Overexpression was induced by adding IPTG (0.1 mM or 0.5 mM); the cultures were grown for another 20 h at 18 °C and harvested (2900×g, 15 min, 4 °C). The cells were resuspended in lysis buffer as shown in Table 4, and then the cells were disrupted by sonication. The sonicated solution was then centrifuged (30 000×g, 4 °C, 60 min) to remove any insoluble parts. The soluble fraction was mixed with Ni‐NTA (nitrilotriacetic acid) and was incubated at 4 °C for 60 min with low speed shaking. The column was then washed by gravity flow with the wash buffer (Table 4). The bound protein was eluted with the elution buffer (Table 4). The fractions were collected and analysed by SDS‐PAGE to select the ones containing the target enzyme. The enzyme solution was desalted with the desalting buffer (Table 4) to remove imidazole. Finally, the enzyme samples were concentrated using Amicon® Ultra‐15 centrifugal filter device (50 kDa cutoff, Millipore). The final enzyme solution was aliquoted, frozen in liquid nitrogen, and stored at −20 °C.

**Table 4:** Buffers for purification.

	NaH_2_PO_4_ [mM]	NaCl [mM]	Glycerol (*w*/*v*)	Imidazole [mM]	pH
lysis buffer	50	100	10 %	10	8
wash buffer	50	100	10 %	20	8
elution buffer	50	100	10 %	250	8
desalting buffer	50	–	10 %	–	7.4

Preparation of DES: DESs ChCl/Gly was prepared by gently heating and stirring with choline chloride and glycerol in a molar ratio of 1 : 2 at a temperature of 80 °C until a clear and homogenous liquid was formed.

Photocatalytic setup: The photoenzymatic decarboxylation reactions catalysed by sCvFAP and ancestral reconstructed proteins were performed at 30 °C in a total volume of 200 μL Tris‐HCl buffer (pH 8.5, 100 mM) containing 30 % DMSO or DES as co‐solvents. The reaction system was added into a transparent glass vial (total volume 5 mL). The vial was sealed and exposed to blue LED light under gentle magnetic stirring with speed 200 rpm. At intervals, aliquots were withdrawn and the reagents were extracted with ethyl acetate (containing 1 mM of cyclohexanol as internal reference) in a 1 : 1 ratio (*v*/*v*). The remaining organic phase was analysed using GC‐FID.

Melting temperature (*T*_m_) determination using ThermoFAD: Melting temperatures for all enzymes were determined using the ThermoFAD method as first described by Forneris et al.^[44]^ While this method does not assess the unfolding equilibrium, it is valuable for establishing the thermostability of a protein. In a real‐time PCR (RT‐PCR) machine (Eppendorf) fitted with a 470–543 nm excitation filter and an SYBR Green emission filter (523–543 nm), 20 μL of 1 mg/mL protein were loaded. A temperature gradient from 10 °C to 95 °C was applied (0.5 °C/min), and fluorescence data were recorded every 0.5 °C. A sigmoidal curve was obtained after plotting the fluorescence against the temperature. The unfolding temperature, *T*_m_, is then determined as the maximum of the derivative of this sigmoidal curve.

Thermostability determination assay with purified enzyme: All enzymatic assays were performed in transparent glass vials sealed with caps having a septum. Reaction mixtures contained 200 μL of each purified enzyme (4 μM), 5 mM fatty acid (50 mM stock in DMSO), and 30 % DMSO as co‐solvent. Thermal stability was measured by incubating the purified enzymes at various temperatures for 10 min with occasional shaking. Activities were determined by assaying the residual activity at 30 °C under standard reaction conditions.

Photoinactivation determination assay with purified enzyme: All purified enzymes were incubated under operational conditions but without substrate for 5, 10 and 15 min. Activities were determined by assaying the residual activity at 30 °C under standard reaction conditions comparing with a control enzymatic reaction without pre‐incubation.

FAD content determination: FAD content of purified enzymes using NanoDrop 2000 (Thermo Fisher Scientific). In particular, the concentrations were determined with the following extinction coefficients at 280 nm: 79300 M^−1^cm^−1^ for sCvFAP, and 61 310 M^−1^cm^−1^ for ANC1 and sANC1 (ProtParam, ExPaSy). All protein signals were corrected for the FAD absorbance at 280 nm using the extinction coefficient 24 300 M^−1^cm^−1^. The concentration of bound FAD was determined at 469 nm using the extinction coefficient 11 300 M^−1^cm^−1^.^[17]^

## Conflict of interest

The authors declare no conflict of interest.

## Supporting information

As a service to our authors and readers, this journal provides supporting information supplied by the authors. Such materials are peer reviewed and may be re‐organized for online delivery, but are not copy‐edited or typeset. Technical support issues arising from supporting information (other than missing files) should be addressed to the authors.

Supplementary

## References

[1] M. P. Johnson , Essays Biochem. 2016, 60, 255–273. 27784776 10.1042/EBC20160016 PMC5264509

[2] T. P. Begley , Acc. Chem. Res. 1994, 27, 394–401.

[3] A. Benjdia , Curr. Opin. Struct. Biol. 2012, 22, 711–720. 23164663 10.1016/j.sbi.2012.10.002

[4] H. M. Wilks , M. P. Timko , Proc. Natl. Acad. Sci. USA 1995, 92, 724–728. 7846042 10.1073/pnas.92.3.724 PMC42692

[5] L. Schmermund , V. Jurkaš , F. F. Özgen , G. D. Barone , H. C. Büchsenschütz , C. K. Winkler , S. Schmidt , R. Kourist , W. Kroutil , ACS Catal. 2019, 9, 4115–4144.

[6] K. Köninger , Á. Gómez Baraibar , C. Mügge , C. E. Paul , F. Hollmann , M. M. Nowaczyk , R. Kourist , Angew. Chem. Int. Ed. 2016, 55, 5582–5585. 10.1002/anie.201601200 27029020

[7] D. Sorigué , B. Légeret , S. Cuiné , S. Blangy , S. Moulin , E. Billon , P. Richaud , S. Brugière , Y. Couté , D. Nurizzo , P. Müller , K. Brettel , D. Pignol , P. Arnoux , Y. Li-Beisson , G. Peltier , F. Beisson , Science 2017, 357, 903–907. 28860382 10.1126/science.aan6349

[8] M. A. Rude , T. S. Baron , S. Brubaker , M. Alibhai , S. B. Del Cardayre , A. Schirmer , Appl. Environ. Microbiol. 2011, 77, 1718–1727. 21216900 10.1128/AEM.02580-10 PMC3067255

[9] Z. Rui , X. Li , X. Zhu , J. Liu , B. Domigan , I. Barr , J. H. D. Cate , W. Zhang , Proc. Natl. Acad. Sci. USA 2014, 111, 18237–18242. 25489112 10.1073/pnas.1419701112 PMC4280606

[10] J. Xu , Y. Hu , J. Fan , M. Arkin , D. Li , Y. Peng , W. Xu , X. Lin , Q. Wu , Angew. Chem. Int. Ed. 2019, 58, 8474–8478; 10.1002/anie.201903165 31033108

[11] M. M. E. Huijbers , W. Zhang , F. Tonin , F. Hollmann , Angew. Chem. Int. Ed. 2018, 57, 13648–13651. 10.1002/anie.201807119 PMC6197046 30106504

[12] W. Zhang , M. Ma , M. M. E. Huijbers , G. A. Filonenko , E. A. Pidko , M. Van Schie , S. De Boer , B. O. Burek , J. Z. Bloh , W. J. H. Van Berkel , W. A. Smith , F. Hollmann , J. Am. Chem. Soc. 2019, 141, 3116–3120. 30673222 10.1021/jacs.8b12282 PMC6385076

[13] W. Zhang , J. H. Lee , S. H. H. Younes , F. Tonin , P. L. Hagedoorn , H. Pichler , Y. Baeg , J. B. Park , R. Kourist , F. Hollmann , Nat. Commun. 2020, 11, 1–8. 32382158 10.1038/s41467-020-16099-7 PMC7206127

[14] I. S. Yunus , J. Wichmann , R. Wördenweber , K. J. Lauersen , O. Kruse , P. R. Jones , Metab. Eng. 2018, 49, 201–211. 30144559 10.1016/j.ymben.2018.08.008

[15] E. Englund , J. Andersen-Ranberg , R. Miao , B. Hamberger , P. Lindberg , ACS Synth. Biol. 2015, 4, 1270–1278. 26133196 10.1021/acssynbio.5b00070 PMC4685428

[16] S. Moulin , B. Légeret , S. Blangy , D. Sorigué , A. Burlacot , P. Auroy , Y. Li-Beisson , G. Peltier , F. Beisson , Sci. Rep. 2019, 9, 13713. 31548626 10.1038/s41598-019-50261-6 PMC6757031

[17] B. Lakavath , T. M. Hedison , D. J. Heyes , M. Shanmugam , M. Sakuma , R. Hoeven , V. Tilakaratna , N. S. Scrutton , Anal. Biochem. 2020, 600, 113749. 32348726 10.1016/j.ab.2020.113749

[18] A. Telzerow , J. Paris , M. Håkansson , J. González-Sabín , N. Ríos-Lombardía , M. Schürmann , H. Gröger , F. Morís , R. Kourist , H. Schwab , K. Steiner , ACS Catal. 2019, 9, 1140–1148.

[19] R. Kazlauskas , Chem. Soc. Rev. 2018, 47, 9026–9045. 30306986 10.1039/c8cs00014j

[20] Y. Xia , W. Cui , Z. Cheng , L. Peplowski , Z. Liu , M. Kobayashi , Z. Zhou , ChemCatChem 2018, 10, 1370–1375.

[21] R. Hoeven, J. Hughes, M. Amer, E. Wojcik, S. Tait, M. Faulkner, I. S. Yunus, S. Hardman, L. Johannissen, G.-Q. Chen, M. Smith, P. Jones, H. Toogood, N. Scrutton, *bioRxiv* preprint **2019**, 10.1101/640474.

[22] M. J. Harms , J. W. Thornton , Curr. Opin. Struct. Biol. 2010, 20, 360–366. 20413295 10.1016/j.sbi.2010.03.005 PMC2916957

[23] R. Merkl , R. Sterner , Biol. Chem. 2016, 397, 1–21. 26351909 10.1515/hsz-2015-0158

[24] M. Wilding , T. S. Peat , S. Kalyaanamoorthy , J. Newman , C. Scott , L. S. Jermiin , Green Chem. 2017, 19, 5375–5380.

[25] D. L. Trudeau , M. Kaltenbach , D. S. Tawfik , Mol. Biol. Evol. 2016, 33, 2633–2641. 27413048 10.1093/molbev/msw138

[26] A. Thomas , R. Cutlan , W. Finnigan , M. van der Giezen , N. Harmer , Commun. Biol. 2019, 2, 1–12. 31799431 10.1038/s42003-019-0677-y PMC6874671

[27] P. Babkova , E. Sebestova , J. Brezovsky , R. Chaloupkova , J. Damborsky , ChemBioChem 2017, 18, 1448–1456. 28419658 10.1002/cbic.201700197

[28] D. Gonzalez , J. Hiblot , N. Darbinian , J. C. Miller , G. Gotthard , S. Amini , E. Chabriere , M. Elias , FEBS Open Bio 2014, 4, 121–127. 10.1016/j.fob.2013.12.006 PMC3907688 24490136

[29] S. Moulin, A. Beyly, S. Blangy, B. Légeret, M. Floriani, A. Burlacot, D. Sorigué, Y. Li-Beisson, G. Peltier, F. Beisson, *bioRxiv* preprint **2020**, 10.1101/2020.06.23.166330.

[30] V. A. Aleksenko , D. Anand , A. Remeeva , V. V. Nazarenko , V. Gordeliy , K.-E. Jaeger , U. Krauss , I. Gushchin , Catalysts 2020, 10, 1072.

[31] S. Costa , A. Almeida , A. Castro , L. Domingues , Front. Microbiol. 2014, 5, 1–20. 24600443 10.3389/fmicb.2014.00063 PMC3928792

[32] D. Esposito , D. K. Chatterjee , Curr. Opin. Biotechnol. 2006, 17, 353–358. 16781139 10.1016/j.copbio.2006.06.003

[33] D. Psimadas , P. Georgoulias , V. Valotassiou , G. Loudos , J. Pharm. Sci. 2012, 101, 2271–2280. 22488174 10.1002/jps.23146

[34] P. Malakar , K. V. Venkatesh , Appl. Microbiol. Biotechnol. 2012, 93, 2543–2549. 22038249 10.1007/s00253-011-3642-3

[35] G. Hannig , S. C. Makrides , Trends Biotechnol. 1998, 16, 54–60. 9487731 10.1016/s0167-7799(97)01155-4

[36] A. L. Larentis , J. F. M. Q. Nicolau , G. dos S Esteves , D. T. Vareschini , F. V. R. de Almeida , M. G. dos Reis , R. Galler , M. A. Medeiros , BMC Res. Notes 2014, 7, 671. 25252618 10.1186/1756-0500-7-671 PMC4190419

[37] D. R. Canchi , A. E. García , Annu. Rev. Phys. Chem. 2013, 64, 273–293. 23298246 10.1146/annurev-physchem-040412-110156

[38] E. R. Parnham , E. A. Drylie , P. S. Wheatley , A. M. Z. Slawin , R. E. Morris , Angew. Chem. 2006, 118, 5084–5088. 10.1002/anie.200600290 16819741

[39] A. P. Abbott , G. Capper , D. L. Davies , R. K. Rasheed , V. Tambyrajah , Chem. Commun. 2003, 70–71. 10.1039/b210714g 12610970

[40] R. Xin , S. Qi , C. Zeng , F. I. Khan , B. Yang , Y. Wang , Food Chem. 2017, 217, 560–567. 27664672 10.1016/j.foodchem.2016.09.012

[41] J. T. Gorke , F. Srienc , R. J. Kazlauskas , Chem. Commun. 2008, 1235–1237. 10.1039/b716317g 18309428

[42] A. K. Schweiger , N. Ríos-Lombardía , C. K. Winkler , S. Schmidt , F. Morís , W. Kroutil , J. González-Sabín , R. Kourist , ACS Sustainable Chem. Eng. 2019, 7, 16364–16370.

[43] A. Karshikoff , L. Nilsson , R. Ladenstein , FEBS J. 2015, 282, 3899–3917. 26074325 10.1111/febs.13343

[44] F. Forneris , R. Orru , D. Bonivento , L. R. Chiarelli , A. Mattevi , FEBS J. 2009, 276, 2833–2840. 19459938 10.1111/j.1742-4658.2009.07006.x

[45] S. K. Chapman , G. A. Reid , Flavoprotein Protocols 1999, (Vol. 131). Springer Science & Business Media.

[46] J. Yang , I. Anishchenko , H. Park , Z. Peng , S. Ovchinnikov , D. Baker , Proc. Natl. Acad. Sci. USA 2020, 117, 1496–1503. 31896580 10.1073/pnas.1914677117 PMC6983395

[47] J. Kyte , R. F. Doolittle , J. Mol. Biol. 1982, 157, 105–132. 7108955 10.1016/0022-2836(82)90515-0

[48] Y. Gumulya , J.-M. Baek , S.-J. Wun , R. E. S. Thomson , K. L. Harris , D. J. B. Hunter , J. B. Y. H. Behrendorff , J. Kulig , S. Zheng , X. Wu , B. Wu , J. E. Stok , J. J. De Voss , G. Schenk , U. Jurva , S. Andersson , E. M. Isin , M. Bodén , L. Guddat , E. M. J. Gillam , Nat. Catal. 2018, 1, 878–888.

[49] C. Notredame , D. G. Higgins , J. Heringa , J. Mol. Biol. 2000, 302, 205–217. 10964570 10.1006/jmbi.2000.4042

[50] D. T. Jones , W. R. Taylor , J. M. Thornton , Bioinformatics 1992, 8, 275–282.

[51] S. Q. Le , O. Gascuel , Mol. Biol. Evol. 2008, 25, 1307–1320. 18367465 10.1093/molbev/msn067

[52] K. Katoh , K. Misawa , K. I. Kuma , T. Miyata , Nucleic Acids Res. 2002, 30, 3059–3066. 12136088 10.1093/nar/gkf436 PMC135756

[53] L. Schrödinger, W. DeLano, **2020**. *PyMOL*, Available at: http://www.pymol.org/pymol.

[54] E. Gasteiger , C. Hoogland , A. Gattiker , S. Duvaud , M. R. Wilkins , R. D. Appel , A. Bairoch in Proteomics Protocols Handbook (Ed.: J. M. Walker ), Humana Press, Totowa, 2005, pp. 571–607.

