# Supplementary material for: A Reconstructed Common Ancestor of the Fatty Acid Photo‐decarboxylase Clade Shows Photo‐decarboxylation Activity and Increased Thermostability
Source: Chembiochem. 2021 Mar 31;22(10):1833–40. doi: 10.1002/cbic.202000851 (PMC8252050; doi:10.1002/cbic.202000851)
Supplement: Supplementary file 1 — Supplementary [file CBIC-22-1833-s001.pdf]

ChemBioChem

Supporting Information

A Reconstructed Common Ancestor of the Fatty Acid Photo-decarboxylase Clade Shows Photo-decarboxylation Activity and Increased Thermostability

Yue Sun, Elia Calderini, and Robert Kourist*

1. DNA sequences used in this study 2
2. Photodecarboxylase CvFAP and ancestral reconstructions expression and purification 5
3. Fluorescence study of CvFAP and ANC1 8
4. Determination of the photodecarboxylation activity for ANC1 with different fatty acid substrates. 9

DNA sequences used in this study.

Table S1: DNA sequences used in this study.

Name	DNA sequence (5 ´to 3 ´)
CvFAP	ATGCAGCTACGGTCGCCGGTGCAGCAGCAGATGCACCAATGGTTGCTTTCCCCGTCAGCAGAGCTGCTG<br>CGCGTTCAGCGATCATAACTACCGGCGCACCGGTCTGACCACCCGGAATTTTCGGAACAACGCTAGCGT<br>CAACAACGCGCAGGCCTTCAACACCGTGAACACGCAGCTGGTTGTCTACCACAGAGCTGCTGTCACCTG<br>CGTTACCCATTTTACAGGTGCCAGTGATAGCGTTGGACGAGTGGATAGAACGACGGATATATTCATCGAT<br>CTGATCATCAGAAACAACGCCGCTACCTGGGAACAGCTCACCATCCAGGTATTCGGACAGAGCAGAGCT<br>ACGCGCAACATCACGTGCCCAATGGATGCCTTTACGCAGGGTAGCCAGATCAGCACCGTCTTTGTCGGT<br>CAGGTAACCTGGTGACAGTTTCGGCGGCGCAAACGGGTCAGCGGATTTAAGACCGACGGAGCCGGTAG<br>ACTGCGGACGGCAAGCGATCAGCTGCATGGTGATGCCGCTCGGCCATTTCAGACCCTGGCTCTGGAATT<br>TAGCAAAACGAACGTAGGTGCTAACACCGTCCGGGTCCAGCGCCATACCTGGAACGAAGCGAACCTGCA<br>GGTCCGGCAGCGCCTGACCCGCGGTACGAACGAAGGCACCGCGATCGCAACCGGTGGAAGTCAGACC<br>GCCACGACCACCCAGCAGGTAGGATGCGATTGCACGTTTACGGATCTGGCCTTTTTCGTTGTAGATGTGA<br>TCAGAAATGGCAATACCGTCGTATTTTTCTTTAACCGGAGCCGCGGTCAGGCACGCCGGCTGATCCTGCA<br>GGTTCTGGCCAACACCAGCCAGGTTGCTAACAACCGGGATGCCGAATTCTTTCAGCTCAGCAGACGGGC<br>CAACGCCGGAATGTTTCAGCAGGAACGGGGTGTGAACAGCACCTGCGCACATGATGACCTCACCACCCG<br>GAGCCAGTTCCGCAGACAGGCGTTCGCCGGTTGGGCCGTCGGTGGAGAATTCAACACCCAGAGCCTGC<br>GCTTTGCCCGCAGCCTGGTCGATGTTGACTTTGGTCACTGCAGCGCCGGTCAGTACCTGCAGGTTGCGA<br>CGACCCAGCACAGGTTTCAGATACTGACGGTACATATCCGCGCGGGTGCCTTTATCCTGCATCACCTGAA<br>AGGTGCCGTAACCGGCGTGGTCATGGCTCCAATCGTTGAAATCGGAGTTCGGGGTAAGACCAACTTCTT<br>CAGCAGCCTTGAAGAAAGCAGTGTGCAGCTGTTTGTTGGTGTAACGCGGGTTTTCCACACGCATCGGGC<br>CGCCGCTGCCATGATAAGCGCCCGGACCGAAGTCCGCGTTGGTTTCCGCCTGGACGAACCAAGACAGA<br>ACGTCTTCGCTGGACCAGCCTTCAACACCCCATGCGTCGTAATCACCCGCCGCACCACGGTGGTACAGA<br>GTGGCGTTAGTCGCGCTGGAACCGCCCAGCAGACGGCCACGCGCCATGTAGATCTGACGTTCCGCAAG<br>CTGTTCCTGCAGTTCAGAGAACAGGTTCCAGTCCAGCGGGGAGCGGAACAGGCGGGTGATCGCCGCCG<br>GAATCTTAACGTCGCGGGAGGTGTTATCCGGGCCTGCTTCCAGAACCAGTACACGTTTGGAACCGTCAG<br>CGCTCAGACGGTTTGCCAGCACGCACGCCGCGGTGCCACCGCCAACCAGGATGTAGTCATATTTCTGAC<br>CCGCCACCGGAGACGAAGAATCGGACAGGACTTTACGGATGTCTTCAACGGCAGACGCGGATTGGAAGT<br>ACAGGTTTTCCTCGATCCCGGCCAGGTTAGCGTCGAGGAACTCTTTCAACTGACCTTTAGACAGTGCACC<br>CACTTTGGTTGCCGCCACTTCACCGTTTTTGAACAGCAGCAGAGTCGGGATACCACGGATGCCATATTTC<br>GGCGCAGTGCCAGGGTTTTGATCGATGTTCAGTTTTGCAACGGTCAGTTTGCCCTGATATTCGTCAGCGA<br>TTTCATCCAGAATCGGGGCGATCATTTTGCACGGACCGCACCACTCTGCCCAGAAATCGACGAGGATCG<br>CCCCGTCCGCTTTGAGTACATCCGTGTCAAAACTGTCGTCAGTCAGGTGAATAATTTTATCGCTCATAGAA<br>GAACCATGGTGATGGTGATGGTGAGAAGATTTCAT
ANC1	ATGATGTCCTCTCGTTCCGTACTGCTGGGTGCTCGTCCAGTTACTCGTGCACCACTGCCAGCTGCAGCTC<br>CTACTCCTGCACGTCGTTCCCAGTCCGGTGCTGCACGTCGTGCTCTGTCTGCTCGTGGTGGTCTGGGCG<br>AAACCCTGCGTAGCGTTGTTAGCGCGTCTAGCTCCCCGGTGGCTTCCGAAAAATACGATTACATTCTGGT<br>AGGCGGCGGTACTGCAGGCTGTGTACTGGCAAATCGTCTGAGCGCTGACGGTTCCAAACGTGTACTGGT<br>TCTGGAAGCTGGCCCAGCGAACAAGTCCCGTGAAGTGCGTATTCCTGCAGGTATTACTCGCCTGTTCCG<br>CAGCCCGCTGGATTGGAATCTGTATAGCGAACTGCAACAGCAGCTGGCCGAACGCGAAATCTATCTGGC<br>TCGTGGTCGTCTGCTGGGCGGTTCTTCTGCCACTAACGCTACCCTGTATCACCGTGGCACTGCTGCAGA<br>CTATGACGCGTGGGGTGTAGAAGGCTGGACCTCCGAAGACGTGCTGTCTTGGTTCGTTAAAGCAGAAAA<br>CAATTACGGCGGTGGTCCGGGTGCATACCATGGTACTGGCGGTCCGATGCGCGTAGAAAATCCGCGCTA<br>TCAGAACCCACTGCATGAGGCTTTCTTCAAGGCAGCGGAAGAAGCAGGTCTGCCGGCTAACCCGGATTT<br>CAACGACTGGTCTCACCCTCAGGCTGGCTACGGTGAGTTCCAGGTGACCCAACGTAAAGGCCAGCGTGC<br>TGACACCTACCGTACCTACCTGAAACCGGTGATGGGTCGTTCTAATCTGCAAGTTCTGACCGGTGCTGCG<br>GTTACCAAAGTTAACATCGAAAAATCTGGCGGTGGTCCTCGTGCACGTGGTGTTGAATTCCAGGCTAACG<br>GTCAGGATGGTGACCGTCACTCTGCCGAACTGGCGCCAGGTGGTGAAGTGCTGATGTGTGCCGGTGCA<br>GTACACACCCCGCATCTGCTGATGCTGTCTGGTATCGGTCCAGCAGCTGAGCTGCGTGAACACGGCATC<br>CCGGTTGTTAGCGACCTGCCGGGTGTTGGCCAGAACCTGCAGGACCATCCGGCTTGTGTCACCGCTGC<br>GCGTGTTAAGGAAAAGTATGAACCGATCTCTGTTACCGACGAGATTTATAACGAAAAAGGCAAAATCCGT<br>CCTCGTGCTGTAGCACAATACCTGCTGGGCGGCCGTGGTCCTCTGACTTCTACTGGTTGCGATCATGGT<br>GCCTTTGTACGCACGGCAGGCCAGGCACAACCTGATCTGCAGATCCGTTTTGTACCTGGTCTGGCTCTG<br>GACCCGGATGGTGTTTCTTCTTATATCGCGTTCGGCAAGCTGAAAAGCCAGGGTCAGAAATGGCCGTCT<br>GGTATTACTCTGCAGCTGCTGGCTGTGCGTCCGAAATCCAAAGGCTCCGTTGGCCTGAAAAGCGCTGAT<br>CCGTTTGCGCCACCGAAAATCGACATTGGTTATCTGACTGACAAGGCTGATCTGGCAACCCTGCGTAACG<br>GCATCAAACTGGCGCGCGAAATTGCTGCCCAGCCTGCACTGGGTGAATACCTGGGCGAAGAACTGTTCC<br>CGGGCGCAGCAGTATCTTCTGACGAGGAAATCGACGAATATATTCGCCGTACTGTGCACAGCGGCAACG<br>CTCTGGTCGGTACCTGCCGTATGGGTAACGCGTCCGATAGCTCTGCGGTGGTTGACAGCGAACTGCGCG<br>TCTTCGGCGTTGAAGGTCTGCGTGTCGTGGATGCAAGCGTTATTCCACGCATTCCAGGTGGCCAGACCG<br>GTGCTCCTACCGTAATGGTTGCGGAACGTGCTGCCGCACTGCTGCGT

ANC2

ATGATGTCTGCACAGTCCGTACTGCTGGGTGCGCGTCCTGTTACCCGTGCTCCTGTACCAGCAGCAGCA
CCGACTCCAGCACGTCGTAGCCAGAGCTCTGCTGCTCGTCGTGCTCTGTCTGCACGTGGTGGCGTAGGT
GAAACGCTGCGTTCTGTCGTTCGTGCATCCTCTTCCCCGGCAGCTTCTGAAAAATACGATTACATCCTGG
TTGGCGGTGGCACCGCTGGTTGTGTTCTGGCAAACCGCCTGAGCGCAGATGGTTCCAAGCGTGTTCTGG
TTCTGGAAGCTGGCCCGGCTAACAAATCCCGCGAAGTTCGTGTTCCGGCGGGTATTACCCGTCTGTTTAA
ATCTCCGCTGGATTGGAACCTGTACTCCGAACTGCAGCAGCAGCTGGCAGACCGTGAAATCTATCTGGC
CCGTGGTCGTCTGCTGGGTGGTTCTTCTGCGACCAACGCTACGCTGTACCACCGTGGTACTGCCGCTGA
TTATGACGCGTGGGGTGTGGAAGGCTGGTCTAGCGAAGATGTTCTGTCCTGGTTCGTGAAAGCTGAAAA
CAATGCAGCCGGTGGTCCTGGTGCTTACCATGGTACCGGTGGTCCAATGCGTGTCGAGAACCCGCGCTA
CCAGAACCCGCTGCATGAAGCGTTTTTCAAAGCTGCGGAAGAAGCAGGTCTGCCGGCAAACCCAGATTT
CAACGACTGGAGCCACCCGCAGGCTGGCTATGGTGAATTCCAGGTCACCCAGAAAAAGGGCCAGCGTG
CTGACGCGTACCGTACGTACCTGAAACCGGCCATGGGTCGTAGCAACCTGCAGGTTCTGACTGGCGCTG
CGGTGACCAAAGTGAACATTGAAAAGAGCGGTGGTGGTGCGCGTGCACGTGGTGTAGAATTCCAAGCTA
ACGGCCAGAACGGTGAACGTCACTCTGCCGAACTGGCACCGGGTGGTGAAGTTCTGATGTGTGCCGGT
GCGGTGCATACTCCACACCTGCTGATGCTGTCTGGCATCGGTCCAGCGGCTGAGCTGCGTGAACACGGT
ATCCCGGTTGTTAGCGACCTGCCGGGTGTTGGTCAGAACCTGCAGGATCACCCGGCATGCGTTACCGCG
GCTCGTGTTAAAGAGAAATATGAACCTATCAGCGTTACCGACGAAATTTACAATGAGAAGGGTAAAATTCG
TGCACGTGCTATCGCTCAATACCTGCTGGGCGGTCGTGGTCCTCTGGCTTCTACTGGTTGTGACCACGG
CGCATTTGTGCGTACCTCCGGCCAGTCTCAACCAGATCTGCAGATTCGTTTTGTCCCGGGTCTGGCTCTG
GATCCAGATGGTGTCTCTTCTTATGTGGCCTTCGGTAAAATGAAAAGCCAGGGTCGTAAATGGCCGTCCG
GCATCACGCTGCAGCTGATCGCTTGTCGCCCTAAGTCCAAGGGTTCTGTTGGTCTGAAATCTGCCGATCC
GTTTGCTCCGCCAAAAATCGATATCGGTTACCTGACTGATAAGGCTGATCTGGCTACCCTGCGTAACGGT
ATCCGCCTGGCACGTGAAATCGCTGCTCAGCCAGCGCTGAGCGAATACCTGGGTGAAGAACTGTTCCCG
GGTGCGGCAGCGTCCTCTGATGAAGAAATCGATGAATATATTCGCCGTACCGTGCACAGCGGTAACGCA
CTGGTGGGTACTTGCAAAATGGGTAACGCATCCGATAGCAACGCCGTTGTAGATTCTGAACTGCGTGTGT
TCGGCGTTGAGGGTCTGCGCGTAGTAGACGCGAGCGTAATCCCGAAAATCCCTGGTGGTCAGACGGGT
GCACCGACCGTTATGGTAGCTGAACGTGCGGCGGCTCTGCTGCGT

ANC3

ATGATGTCTCGCATTGCAGTACTGCTGTCTACCAGCCCATCCCTGATTGCAGCTTCTTCTAGCAGCTCTC
CAGCTCCGGCACGTGCTCCAGCATCTAGCTCTGCGCGTCGTGCTCTGTCTCTGCGTGGTGGTGCCGGTA
CTGTTGCGGCAAGCGTTCGTCGTGCTGCACCGTCTCCGGTAGCGTCTACTACCTATGATTACATTATCGT
CGGTGGCGGCACTGCAGGCTGTGTTCTGGCAAACCGTCTGTCCGCGGATGGCTCTAAACGTGTTCTGGT
TCTGGAGGCTGGCGGTTCTGCCTCTCGCTCTCTGTATGTTCGTATCCCGGCTGGTATCACCCGTCTGTTT
CGTTCTGCATATGACTGGAATTTCAGCACCGAACCGGAGCAGGGCCTGAATGGTCGTGAAGTTTACCTGT
GCCGCGGTAAAGCTCTGGGCGGTAGCTCTTGTACTAACGTCATGCTGTACCATCGTGGTTCTGCCGCAG
ACTACGACGCTTGGGCTGCTGCTGGTGCTGAAGGCTGGGGTCCAGAGGATGTTCTGCCGTATTTTAAGA
AAGCAGAAAACAACGCTGCGGGTGGCGCAAATCAGTACCATGGTTCCGGTGGTCCGCTGGCAGTTGAAG
ATGTACGCTACCAGAACCCGCTGTCCAAGGCGTTTCTGGAAGCAGCCGAAGAAGCAGGTCTGCGTGCAA
ACCCGGACTTCAATGACTGGTCTCACCCACAAGACGGTTACGGCCGTTTCCAGGTAACTCAGCGTAACG
GCCGTCGTTGTTCTGCCGCAACTGCATATCTGCGTGCAGCTCGCGGTCGTTCTAACCTGCATGTTGTTAC
CGGCGCTGCTGCCACCCGTGTAACTCTGGAAGGTTCTGGTGATGGTGCTGGTGGTGGTGGTGGCGGTA
AAACTCGTCCATGGACTGGTCCGGCCGTAACCGGTCAAGCTGGTCCTCGTGCTACGGGCGTTGAATTCA
TTGGTGCAGATGGTGACCGTCGTACCGCCGAAGTCTCCTCTGCACGTCTGGCACAGGGTGGCGAGGTTC
TGCTGTGTGCGGGTGCAGTTCATTCCCCGCAGCTGCTGATGCTGTCCGGCATTGGCCCGGCTGCACATC
TGCGTGAAGTAGGTATCCCGGTTGTGGCTGATCTGCCGGGTGTTGGTCAGAACCTGCAGGATCATCCGG
CAGTCGTGGTAAGCTATCGCAGCAAAAAAGGTGTGAGCGTGACCGATGAAATTCGCCTGTTCGGTACTTC
TAAAACCAACCCGATGGCAGTTCTGCAGTGGCTGCTGTTCGGTCGTGGCCCACTGACTTCTCCTGGCTG
CGATCACGGCGGCTTTGTGCGTACCTCTCCATCTCTGGAGCAGCCGGATCTGCAGATCCGTTTCGTACC
TGCTCGTGCTCTGGATCCGGATGGTATGTCTACTTACACGGAGTTCGGCACTGCGGCGAAGCGTCTGTC
TGGTTTCACTCTGCAGTCTGTTGCGTGTCGTCCGAAAAGCAAAGGTCGTGTTCGTCTGGCTTCTGCGGAT
CCGTTCGCCAAACCGATGATTGAAGGCGGTTACCTGTCTGATGAAGCAGATCTGGCGACCCTGCGTAAT
GGTATTCGTCTGGGTCGTGAGCTGGCTGCCCAACCAGCATTTGGCGAATACCGTGGCGAGGAAGTATTC
CCGGGTGCTGCAGTTCAGTCCGATGAGGACATCGACGCCTATATCCGTAACACCGTACACACCGCTAAC
GCTCTGGTAGGTACCTGTCGTATGGGTAATGCGAGCGACCGTTCCGCAGTTGTAGACCCAGAACTGCGC
GTAATTGGTGTTGGCGGTCTGCGCGTTGTAGACGCGTCCGTAATGCCTACCATCCCGGGTGGTCAGACT
GGTGCTCCGACCGTAATGCTGGCTGAACGTGCTGCGGATCTGGTCCGT

sANC1

ATGGTGGTGGTGGTGGTGGTGCTCGAGACGCAGCAGTGCGGCAGCACGTTCCGCAACCATTACGGTAG
GAGCACCGGTCTGGCCACCTGGAATGCGTGGAATAACGCTTGCATCCACGACACGCAGACCTTCAACGC
CGAAGACGCGCAGTTCGCTGTCAACCACCGCAGAGCTATCGGACGCGTTACCCATACGGCAGGTACCGA
CCAGAGCGTTGCCGCTGTGCACAGTACGGCGAATATATTCGTCGATTTCCTCGTCAGAAGATACTGCTGC
GCCCGGGAACAGTTCTTCGCCCAGGTATTCACCCAGTGCAGGCTGGGCAGCAATTTCGCGCGCCAGTTT
GATGCCGTTACGCAGGGTTGCCAGATCAGCCTTGTCAGTCAGATAACCAATGTCGATTTTCGGTGGCGCA
AACGGATCAGCGCTTTTCAGGCCAACGGAGCCTTTGGATTTCGGACGCACAGCCAGCAGCTGCAGAGTA
ATACCAGACGGCCATTTCTGACCCTGGCTTTTCAGCTTGCCGAACGCGATATAAGAAGAAACACCATCCG
GGTCCAGAGCCAGACCAGGTACAAAACGGATCTGCAGATCAGGTTGTGCCTGGCCTGCCGTGCGTACAA

	AGGCACCATGATCGCAACCAGTAGAAGTCAGAGGACCACGGCCGCCCAGCAGGTATTGTGCTACAGCAC<br>GAGGACGGATTTTGCCTTTTTCGTTATAAATCTCGTCGGTAACAGAGATCGGTTCATACTTTTCCTTAACA<br>CGCGCAGCGGTGACACAAGCCGGATGGTCCTGCAGGTTCTGGCCAACACCCGGCAGGTCGCTAACAAC<br>CGGGATGCCGTGTTCACGCAGCTCAGCTGCTGGACCGATACCAGACAGCATCAGCAGATGCGGGGTGT<br>GTACTGCACCGGCACACATCAGCACTTCACCACCTGGCGCCAGTTCGGCAGAGTGACGGTCACCATCCT<br>GACCGTTAGCCTGGAATTCAACACCACGTGCACGAGGACCACCGCCAGATTTTTCGATGTTAACTTTGGT<br>AACCGCAGCACCGGTCAGAACTTGCAGATTAGAACGACCCATCACCGGTTTCAGGTAGGTACGGTAGGT<br>GTCAGCACGCTGGCCTTTACGTTGGGTCACCTGGAACTCACCGTAGCCAGCCTGAGGGTGAGACCAGTC<br>GTTGAAATCCGGGTTAGCCGGCAGACCTGCTTCTTCCGCTGCCTTGAAGAAAGCCTCATGCAGTGGGTT<br>CTGATAGCGCGGATTTTCTACGCGCATCGGACCGCCAGTACCATGGTATGCACCCGGACCACCGCCGTA<br>ATTGTTTTCTGCTTTAACGAACCAAGACAGCACGTCTTCGGAGGTCCAGCCTTCTACACCCCACGCGTCA<br>TAGTCTGCAGCAGTGCCACGGTGATACAGGGTAGCGTTAGTGGCAGAAGAACCGCCCAGCAGACGACC<br>ACGAGCCAGATAGATTTCGCGTTCGGCCAGCTGCTGTTGCAGTTCGCTATACAGATTCCAATCCAGCGG<br>GCTGCGGAACAGGCGAGTAATACCTGCAGGAATACGCACTTCACGGGACTTGTTCGCTGGGCCAGCTTC<br>CAGAACCAGTACACGTTTGGAACCGTCAGCGCTCAGACGATTTGCCAGTACACAGCCTGCAGTACCGCC<br>GCCTACCAGAATGTAATCGTATTTTTCGGAAGCCAT

2. Photodecarboxylase CvFAP and ancestral reconstructions expression and purification.

The sequence of CvFAP was cloned into plasminds pASK and pBAD. Culture of *E.coli* strain Top 10 harbouring pBAD-CvFAP-GFP were grown in LB-medium with 100 μg/ml ampicillin in shaking flasks (37°C until induction). The expression was induced with L-arabinose at concentration of 0.02% at OD600 nm of 0.8 at 18°C. 20h after induction (18°C), the cells were harvested.

The expression and purification of the proteins were performed as described in experienmantal section. SDS-PAGE analysis with Comassie staining showed that the ancestral proteins (ANC1; ANC2; and ANC3) bands are identifiable at approximately 65.0 KDa, the short ANC1 was 60.0KDa, in agreement with the theoretical mass.

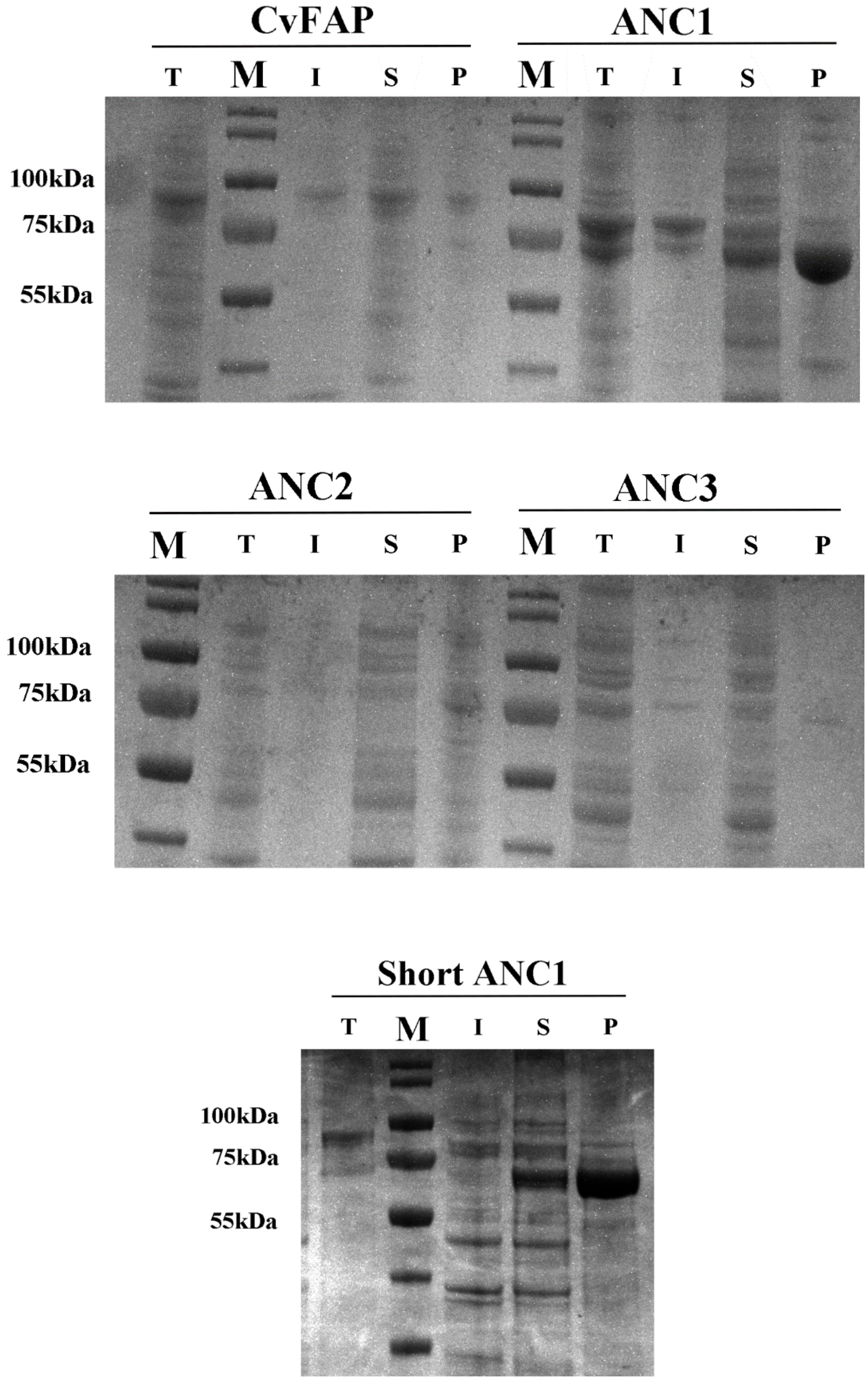

Figure S1. Expression of CvFAP and ancestral sequence reconstructions. Lane M, molecular mass marker; Lane T, total cell lysate; Lane S, soluble fraction of cell lysate; Lane I, insoluble fraction. Molecular weight of the CvFAP: 77 kDa; ANC1: 65 kDa; ANC2: 65 kDa; ANC3: 65 kDa; short ANC1: 60 kDa.

Table S2. Purification yields of CvFAP, ANC1, ANC2, ANC3 and sANC1 enzyme. (mg/L cultivation volume)

Expression vector and IPTG concentration	Purification yield	Expression vector and IPTG concentration	Purification yield
pET28a-sCvFAP (0.1 mM IPTG)	2.34 ± 0.2 mg/L	pET28a-sCvFAP (0.5 mM IPTG)	13.3 ± 2.3 mg/L
pET28a-ANC1 (0.1 mM IPTG)	24.09 ± 1.0 mg/L	pET28a-ANC1 (0.5 mM IPTG)	32.2 ± 2.5 mg/L
pET28a-ANC2 (0.1 mM IPTG)	1.4 ± 0.2 mg/L	pET28a-ANC3 (0.1 mM IPTG)	2.6 ± 0.7 mg/L
pET28a-sANC1 (0.1 mM IPTG)	45.72 mg/L	pET28a-sANC1 (0.5 mM IPTG)	33.5 ± 4.7 mg/L
pASK-CvFAP (200 μg/L Anhydrotetracycline)	15.92 mg/L	pBAD-CvFAP (0.1% Arabinose)	16.94 mg/L

*Purified proteins concentrations were determined through a BCA Assay, using BSA as a standard.

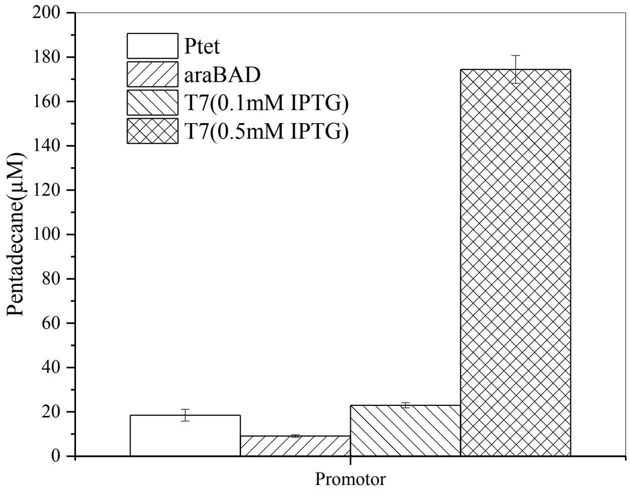

Figure S2. CvFAP decarboxylation activity when induced under the control of different promoters. Condition: Purified enzyme=4 μM, palmitic acid=5 mM, Tris-HCl (pH 8.5, 100 mM), 30% DMSO, illumination with blue light.

3. Fluorescence study of CvFAP and ANC1.

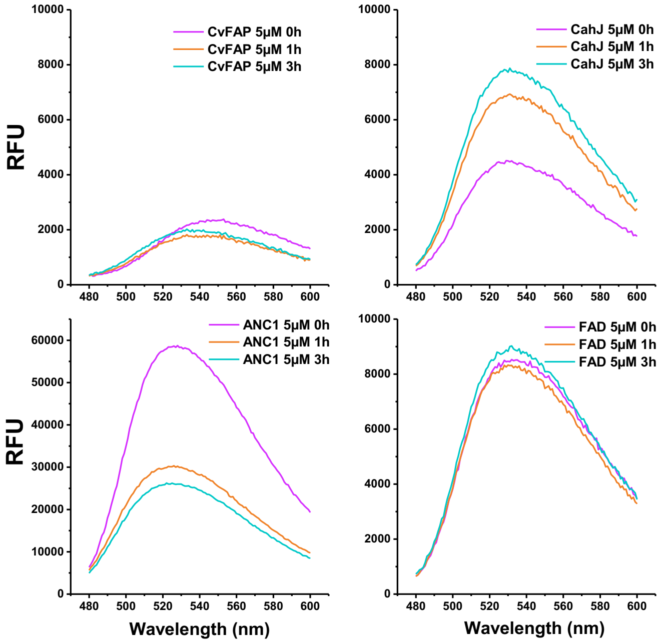

Figure S3. Time-resolved emission spectroscopy of CvFAP, ANC1 CahJ as positive control and FAD as negative control. CvFAP and ANC1 showed opposite trend with the FAD signal decreasing

in contrast to the positive control, FAD remained relatively constant. All measurements were performed using 5 μM of purified enzyme in Tris-HCl buffer pH 8.5 (100 mM); 30°C. Fluorimeter setting are as follow: λex=450 nm, λem=480-600 nm, Gain=100 nm.

4. Determination of the photodecarboxylation activity for ANC1 with different fatty acid substrates at different wave lengths.

Substrates scope for middle chain fatty acid of CvFAP and ancestral enzymes applied as whole cells catalysts. The photodecarboxylation activity study was performed with C12, C14 and C16 as substrates. The vials were sealed and exposed to the LED light with changing wavelength every 5 seconds. CvFAP was selected as control. Analysis of the product profile via GC-MS showed that ANC1 reached a conversion similar to CvFAP. However, ANC2 and ANC3 showed no activity even with different wavelength.

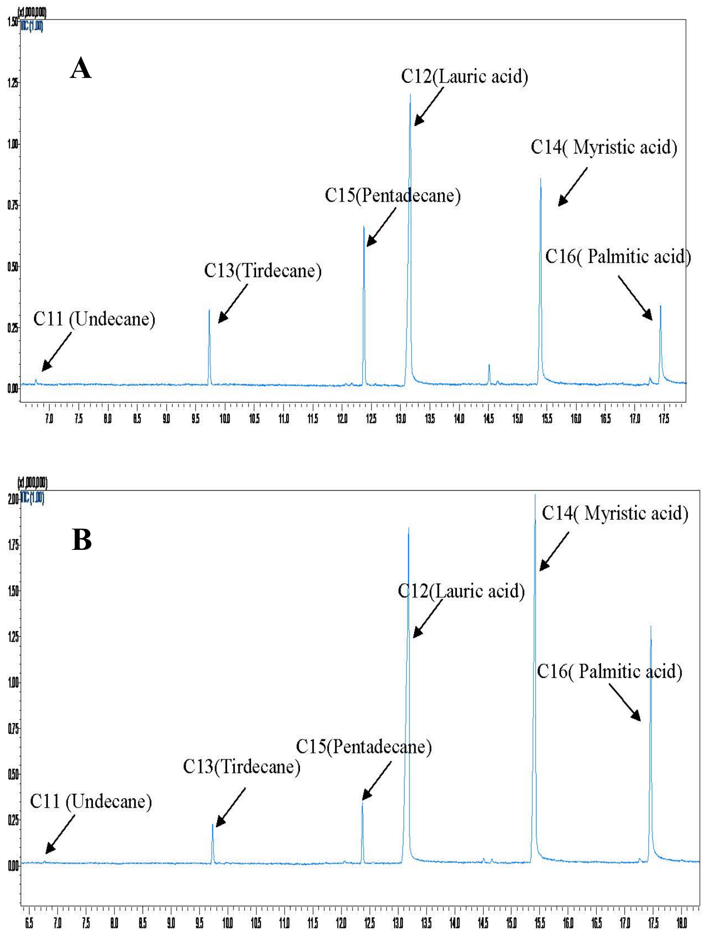

Figure S4. GC chromatogram from the MS channel. The peaks correspond to the observed gaseous products at different retention times. A: CvFAP; B: ANC1.

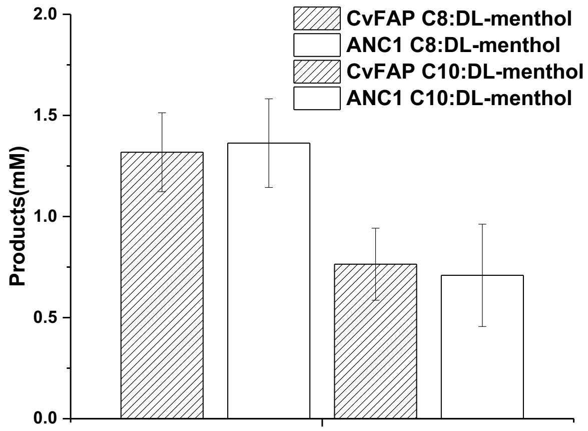

Figure S5. The photoenzymatic decarboxylation reactions catalysed by CvFAP and ANC1 were performed with gentle magnetic stirring at 30 °C in a total volume of 1.0 mL Tris-HCl buffer (pH

8.5, 250 mM) containing DES (Deep Eutectic Solvents) for 20 hours. The final conditions for these reactions were: [fatty acid] =100 mM and [lyophilized whole cells] =10 mg.
*DES: C8:DL-menthol (molar ratio:1:1); C10:DL-menthol (molar ratio:1:1).

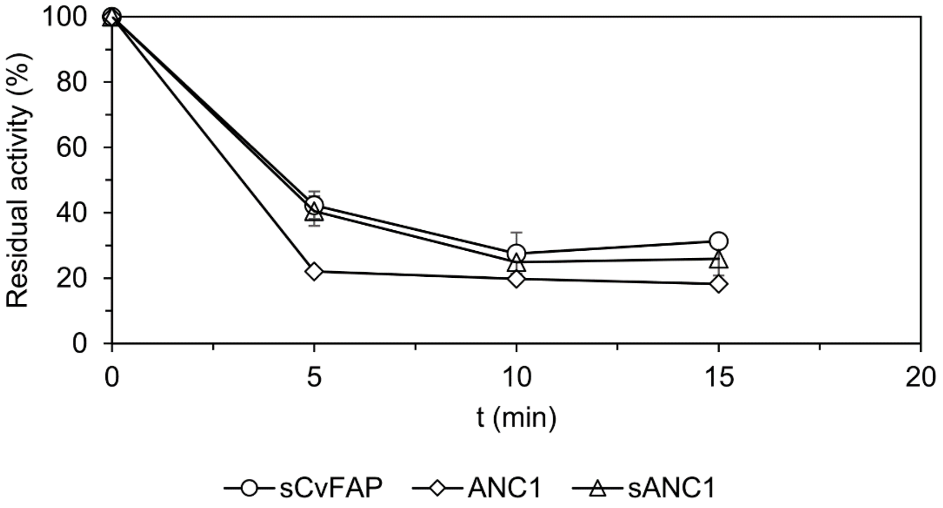

Figure S6. Comparison of sCvFAP, ANC1 and sANC1 residual activities after being exposed to light at 455 nm for the stated amount of time.

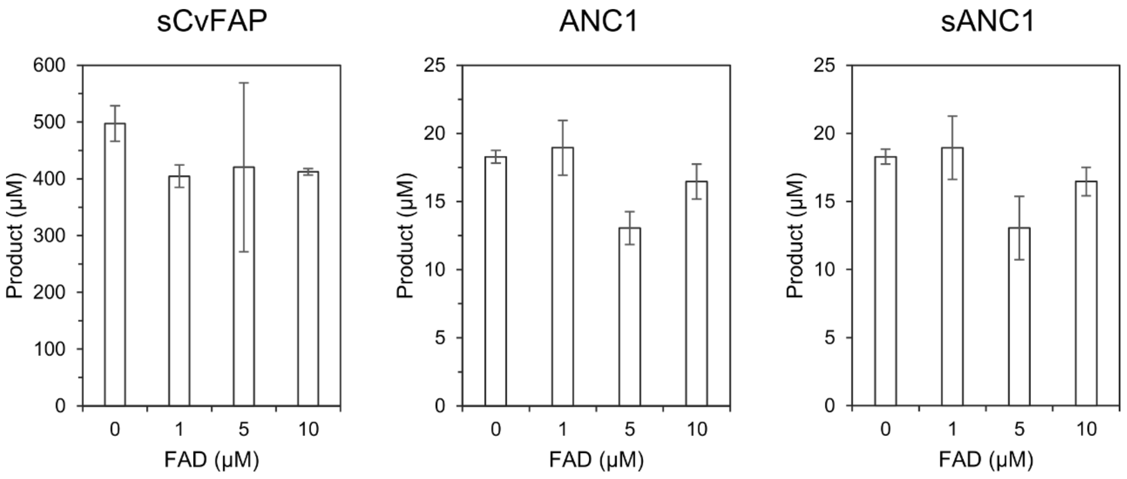

Figure S7. Comparison of sCvFAP, ANC1 and sANC1 product production at different external FAD concentration. In all reactions the enzyme concentration was kept at 5 μM and reactions were performed for 1 h.

Figure S8. Multiple sequence alignment of sCvFAP, ANC1 ANC2 and ANC3 using Clustal Omega.

```
ANC3        ------------------------------------------------------------    0
sCvFAP      MKSSHHHHHHGSSMSDKIIHLTDDSFDTDVLKADGAILVDFWAEWCGPCKMIAPILDEIA    60
ANC1        ------------------------------------------------------------    0
ANC2        ------------------------------------------------------------    0


ANC3        -------------------------MMSRIAVL-LSTSPSL----IAASSSSSPAPARAP    30
sCvFAP      DEYQGKLTVAKLNIDQNPGTAPKYGIRGIPTLLLFKNGEVAATKVGALSKGQLKEFLDAN    120
ANC1        -------------------------MMSSRSVL-LGARPVTRAPLPAAAP----TPARRS    30
ANC2        -------------------------MMSAQSVL-LGARPVTRAPVPAAAP----TPARRS    30
                                     : .  ::* :           * :

ANC3        ASSSARRALSLRGGAGTVAASVRRAAPSPVASTTYDYIIVGGGTAGCVLANRLSADGSKR    90
sCvFAP      LAGIEENLYFQSASAVEDIRKVLSDSSSPVAGQKYDYILVGGGTAACVLANRLSADGSKR    180
ANC1        QSGAARRALSARGGLGETLRSVVSASSSPVASEKYDYILVGGGTAGCVLANRLSADGSKR    90
ANC2        QSSAARRALSARGGVGETLRSVVRASSSPAASEKYDYILVGGGTAGCVLANRLSADGSKR    90
             :.  ..     ..      .*   : **.*. .****:******.**************

ANC3        VLVLEAGGSASRSLYVRIPAGITRLFRSAYDWNFSTEPEQGLNGREVYLCRGKALGGSSC    150
sCvFAP      VLVLEAGP-DNTSRDVKIPAITRLFRSPLDWNLFSELQEQLAERQIYMARGRLLGGSSA    239
ANC1        VLVLEAGP-ANKSREVRIPAGITRLFRSPLDWNLYSELQQQLAEREIYLARGRLLGGSSA    149
ANC2        VLVLEAGP-ANKSREVRVPAGITRLFKSPLDWNLYSELQQQLADREIYLARGRLLGGSSA    149
            *******   . *  *::**.*****:*  ***: :* :: *  *::*:.**: *****.

ANC3        TNVMLYHRGSAADYDAWAAAGAEGWGPEDVLPYFKKAENNAAGGANQYHGSGGPLAVEDV    210
sCvFAP      TNATLYHRGAAGDYDAWG---VEGWSSEDVLSWFVQAETNADFGPGAYHGSGGPMRVENP    296
ANC1        TNATLYHRGTAADYDAWG---VEGWTSEDVLSWFVKAENNYGGGPGAYHGTGGPMRVENP    206
ANC2        TNATLYHRGTAADYDAWG---VEGWSSEDVLSWFVKAENNAAGGPGAYHGTGGPMRVENP    206
            **. *****:*.*****.   .***  **** :* :**.*   * . ***:***: **:

ANC3        RYQN-PLSKAFLEAAEEAGLRANPDFNDWSHPQDGYGRFQVTQRNGRRCSAATAYLRAAR    269
sCvFAP      RYTNKQLHTAFFKAAEEVGLTPNSDFNDWSHDHAGYGTFQVMQDKGTRADMYRQYLKPVL    356
ANC1        RYQN-PLHEAFFKAAEEAGLPANPDFNDWSHPQAGYGEFQVTQRKGQRADTYRTYLKPVM    265
ANC2        RYQN-PLHEAFFKAAEEAGLPANPDFNDWSHPQAGYGEFQVTQKKGQRADAYRTYLKPAM    265
            ** *  *  **::****.**  * ******* : *** *** * :* *..    **: .

ANC3        GRSNLHVVTGAAATRVTLEGSGDGAGGGGGGKTRPWTGPAVTGQAGPRATGVEFIGADGD    329
sCvFAP      GRRNLQVLTGAAVTKVNIDQAAGK----------------------AQALGVEFSTD---    391
ANC1        GRSNLQVLTGAAVTKVNIEKSGGG----------------------PRARGVEFQAN---    300
ANC2        GRSNLQVLTGAAVTKVNIEKSGGG----------------------ARARGVEFQAN---    300
            ** **:*:****.*:*.:: :..                        :* ****

ANC3        RRTAEVSSARLAQGGEVLLCAGAVHSPQLLMLSGIGPAAHLREVGIPVVADLPGVGQNLQ    389
sCvFAP      GPTGERLSAELAPGGEVIMCAGAVHTPFLLKHSGVGPSAELKEFGIPVVSNLAGVGQNLQ    451
ANC1        GQDGDRHSAELAPGGEVLMCAGAVHTPHLLMLSGIGPAAELREHGIPVVSDLPGVGQNLQ    360
ANC2        GQNGERHSAELAPGGEVLMCAGAVHTPHLLMLSGIGPAAELREHGIPVVSDLPGVGQNLQ    360
               .:  **.** ****::******:* **  **:**:*.*:* *****::* *******

ANC3        DHPAVVVSYRSKK---GVSVTDEIRLFGTSKTNPMAVLQWLLFGRGPLTSPGCDHGGFVR    446
sCvFAP      DQPACLTAAPVKEKYDGIAISDHIY-NEKGQIRKRAIASYLLGGRGGLTSTGCDRGAFVR    510
ANC1        DHPACVTAARVKEKYEPISVTDEIY-NEKGKIRPRAVAQYLLGGRGPLTSTGCDHGAFVR    419
ANC2        DHPACVTAARVKEKYEPISVTDEIY-NEKGKIRARAIAQYLLGGRGPLASTGCDHGAFVR    419
            *:** :.:   *:    ::::*.*    ..: .  *:.:**  *** *:* ***:*.***

ANC3        TSPSLEQPDLQIRFVPARALDPDGMSTYTEFGTA----AKRLSGFTLQSVACRPKSKGRV    502
sCvFAP      TAG-QALPDLQVRFVPGMALDPDGVSTYVRFAKFQSQGLKWPSGITMQLIACRPQSTGSV    569
ANC1        TAG-QAQPDLQIRFVPGLALDPDGVSSYIAFGKLKSQGQKWPSGITLQLLAVRPKSKGSV    478
ANC2        TSG-QSQPDLQIRFVPGLALDPDGVSSYVAFGKMKSQGRKWPSGITLQLIACRPKSKGSV    478
            *:     ****:****. ******:*:*  *..      *  **:*:* :* **:*.* *

ANC3        RLASADPFAKPMIEGGYLSDE--ADLATLRNGIRLGRELAAQPAFGEYRGEEVFPGAAVQ    560
sCvFAP      GLKSADPFAPPKLSPGYLTDKDGADLATLRKGIHWARDVARSSALSEYLDGELFPGSGVV    629
ANC1        GLKSADPFAPPKIDIGYLTDK--ADLATLRNGIKLAREIAAQPALGEYLGEELFPGAAVS    536
ANC2        GLKSADPFAPPKIDIGYLTDK--ADLATLRNGIRLAREIAAQPALSEYLGEELFPGAAAS    536
             * ****** * :. ***:*:  *******:**: .*::* . *:.** . *:***:..

ANC3        SDEDIDAYIRNTVHTANALVGTCRMGNASDRSAVVDPELRVIGVGGLRVVDASVMPTIPG    620
sCvFAP      SDDQIDEYIRRSIHSSNAITGTCKMGNAGDSSSVVDNQLRVHGVEGLRVVDASVVPKIPG    689
ANC1        SDEEIDEYIRRTVHSGNALVGTCRMGNASDSSAVVDSELRVFGVEGLRVVDASVIPRIPG    596
ANC2        SDEEIDEYIRRTVHSGNALVGTCKMGNASDSNAVVDSELRVFGVEGLRVVDASVIPKIPG    596
            **::** ***.::*:.**:.***:****.* .:*** :*** ** *********:* ***

ANC3        GQTGAPTVMLAERAADLVR-----------------    639
sCvFAP      GQTGAPVVMIAERAAALLTGKATIGASAAAPATVAA    725
ANC1        GQTGAPTVMVAERAAALLR-----------------    615
ANC2        GQTGAPTVMVAERAAALLR-----------------    615
            ******.**:***** *:
```

Table S3. Specific activities measured for sCvFAP, ANC1 and sANC1 produced at two different IPTG concentrations. Specific activity is given as units per mg of enzyme containing FAD.

Enzyme	IPTG concentration (mM)	U/mg FAD loaded enzyme
sCvFAP	0.1	6.14 ± 0.42
ANC1	0.1	3.62 ± 0.09
sANC1	0.1	4.77 ± 0.33
sCvFAP	0.5	46.61 ± 4.85
ANC1	0.5	4.72 ± 0.23
sANC1	0.5	6.91 ± 0.5
